# A bimodal switch in global protein translation coupled to eIF4H relocalisation during advancing cell-cell transmission of herpes simplex virus

**DOI:** 10.1371/journal.ppat.1007196

**Published:** 2018-07-20

**Authors:** Catherine Su Hui Teo, Peter O’Hare

**Affiliations:** Section of Virology, Faculty of Medicine, Imperial College London, St Mary’s Medical School, London, United Kingdom; University of Alberta, CANADA

## Abstract

We used the bioorthogonal protein precursor, homopropargylglycine (HPG) and chemical ligation to fluorescent capture agents, to define spatiotemporal regulation of global translation during herpes simplex virus (HSV) cell-to-cell spread at single cell resolution. Translational activity was spatially stratified during advancing infection, with distal uninfected cells showing normal levels of translation, surrounding zones at the earliest stages of infection with profound global shutoff. These cells further surround previously infected cells with restored translation close to levels in uninfected cells, reflecting a very early biphasic switch in translational control. While this process was dependent on the virion host shutoff (vhs) function, in certain cell types we also observed temporally altered efficiency of shutoff whereby during early transmission, naïve cells initially exhibited resistance to shutoff but as infection advanced, naïve target cells succumbed to more extensive translational suppression. This may reflect spatiotemporal variation in the balance of oscillating suppression-recovery phases. Our results also strongly indicate that a single particle of HSV-2, can promote pronounced global shutoff. We also demonstrate that the vhs interacting factor, eIF4H, an RNA helicase accessory factor, switches from cytoplasmic to nuclear localisation precisely correlating with the initial shutdown of translation. However translational recovery occurs despite sustained eIF4H nuclear accumulation, indicating a qualitative change in the translational apparatus before and after suppression. Modelling simulations of high multiplicity infection reveal limitations in assessing translational activity due to sampling frequency in population studies and how analysis at the single cell level overcomes such limitations. The work reveals new insight and a revised model of translational manipulation during advancing infection which has important implications both mechanistically and with regards to the physiological role of translational control during virus propagation. The work also demonstrates the potential of bioorthogonal chemistry for single cell analysis of cellular metabolic processes during advancing infections in other virus systems.

## Author summary

All viruses reprogram protein synthesis within infected cells for the production of their own proteins and for suppression of host antiviral responses. On the other hand, cells also modulate translation to suppress virus replication. Thus, global protein synthesis in infected cells represents the temporally regulated interplay of multiple translational processes. While protein synthesis has been generally studied by methods that investigate the average behaviour in cell populations, information at the individual cell level is also critical for a true understanding of the processes governing infection. We used novel techniques in chemical biology that enables spatial analysis of translation levels at the single cell level, examining protein synthesis during cell-to-cell spread of herpes simplex virus. This work leads to a new interpretation of previous models of translational suppression that would not be gained from population studies and demonstrates the broad potential of chemical biology for spatial and biochemical studies of virus infection.

## Introduction

Much of our understanding of the molecular mechanisms operating during virus infection comes from population studies. The classic single-step virus growth cycle, the identification and characterisation of virus encoded transcripts and proteins, and the associated mechanisms governing temporal regulation of their production and turnover have been founded on population studies of infected cells in culture systems [1]. However, it is becoming clear in many fields that while analysis of the average behaviour in total infected cell populations is vital, information at the individual cell level is also critical for a true understanding of the processes governing the outcomes of infection. Such analyses may support and refine conclusions from population studies, but can also yield results which are not accounted for in population studies and provide conceptually new mechanistic insight [2–5]. In this regard, while much effort has focussed on analysis of levels and variations in transcription patterns at the single cell level, we know much less with regard to protein synthesis. All viruses manipulate the host cell translational apparatus to promote the synthesis of their proteins and to supress cellular antiviral responses. At the same time, cells modulate both their qualitative translational output and their translational apparatus in the attempt to suppress virus replication [6–15]. Thus, overall infected cell protein synthesis results from a complex and temporally regulated interplay of multiple distinct translational objectives for the host and virus, in addition to selective controls on the abundance and localisation of individual protein species. However, global protein synthesis has been almost universally studied by population methods such as gel electrophoresis and autoradiography, Western blotting or mass spectrometry, potentially masking dynamic and diverse individual cell behaviour [16–21]. A complete understanding of infected cell protein metabolism requires a parallel approach to spatial aspects of protein synthesis and temporal alterations in these processes at the single cell level during the progression of infection. Traditional steady-state analysis using antibodies, or gene fusion to fluorescent proteins, provide powerful tools for the investigation of individual proteins [22–24]. However, global spatial analysis requires a different approach. Recent advances in bioorthogonal chemistry [25] have facilitated the development of new techniques based on the in vivo incorporation of metabolic precursors containing designed chemical end-groups. Subsequent highly specific covalent bond-forming reactions, commonly termed “click chemistry”, then link the macromolecular products incorporating these precursors to capture reagents via a dedicated, paired end-group [26–29]. The chemical pairs most routinely used are the azide- and alkyne moieties which are small, inert and can be introduced to a variety of precursors [28, 30–33].

Thus, for protein synthesis it is possible to label newly synthesised proteins over a specified timeframe using the methionine analogues homopropargylglycine (HPG) or azidohomoalanine (AHA), and then covalently couple those de novo synthesised proteins to fluorescent capture reagents. This enables analysis either biochemically e.g. by SDS-PAGE and in-gel fluorescence, or spatially to simultaneously visualise the overall levels and localisation of the “translatome” by microscopy [32, 33]. We recently used these techniques to provide the first spatial and kinetic analysis of bulk newly synthesised proteins during a single-step replication cycle of herpes simplex virus (HSV), providing new insight into protein synthesis and trafficking, including the formation of novel nuclear depots into which newly synthesised host and viral proteins trafficked [34]. Here we visualise global protein translation during HSV cell-to-cell transmission. Current models indicate that HSV progressively suppresses infected cell translation, by multiple processes but particularly involving the structural component vhs, an RNase that is the product of the UL41 gene [14, 35–39]. Our results agree with the considerable data from several laboratories that vhs is a key determinant of translational suppression during HSV infection. However from spatial analysis at the single cell level during virus spread we now demonstrate a very early biphasic switch, combining efficient suppression of translation with subsequent recovery to normal levels and show that such oscillations would be averaged out to the unimodal kinetic that is currently proposed from population studies. We also show a highly correlated relocalisation to the nucleus of eIF4H, a factor known to interact with vhs [40, 41] in cells exhibiting translational suppression. Moreover, we show that a single particle of HSV-2 was sufficient to promote translational shutdown, a result not approachable by current methods. Together with other results, this work has significant implications for our understanding of the mechanism(s) and role of translational control, leading to a new interpretation of that would not be gained from population studies. The work also demonstrates the broad potential of chemical biology for spatial and biochemical studies of virus infection, applicable to other viral and indeed bacterial systems.

## Results

### Spatial analysis of global protein synthesis during HSV cell-cell transmission

A schematic indicating the principle of HPG incorporation into proteins and then ligation with azide-linked fluorophores is summarised in S1a Fig. Much accumulated data has demonstrated that HPG has no effect on global rates of protein synthesis nor protein degradation [42–48]. In examining parameters for virus transmission studies, we first pulsed cells with HPG for 30 min at 1 hr after high multiplicity infection with HSV (MOI 10) or mock infection and analysed protein synthesis by SDS-PAGE and in-gel fluorescence (S1b Fig). As a control we also included cycloheximide (CHX, 100 μg/ml) to block de novo protein synthesis. The results demonstrate efficient labeling of uninfected cell proteins (lane 7) and almost complete inhibition by CHX (lane 5), demonstrating as expected that HPG incorporation reflects de novo protein translation. In population experiments such as this (which analyse the total cells in the sample), we observed very little change at this early time in overall levels of translation in HSV infected cells compared to uninfected cells (c.f. lanes 7 and 8). Using a 30 min labeling interval as a benchmark, we examined different labeling intervals to optimise spatial analysis of translation in individual cells (S1c Fig). Although shorter pulse times (5–10 min) gave a detectable signal, we selected 30 min as the standard interval for all subsequent analyses since it gave a good signal and dynamic range.

**Fig 1:**
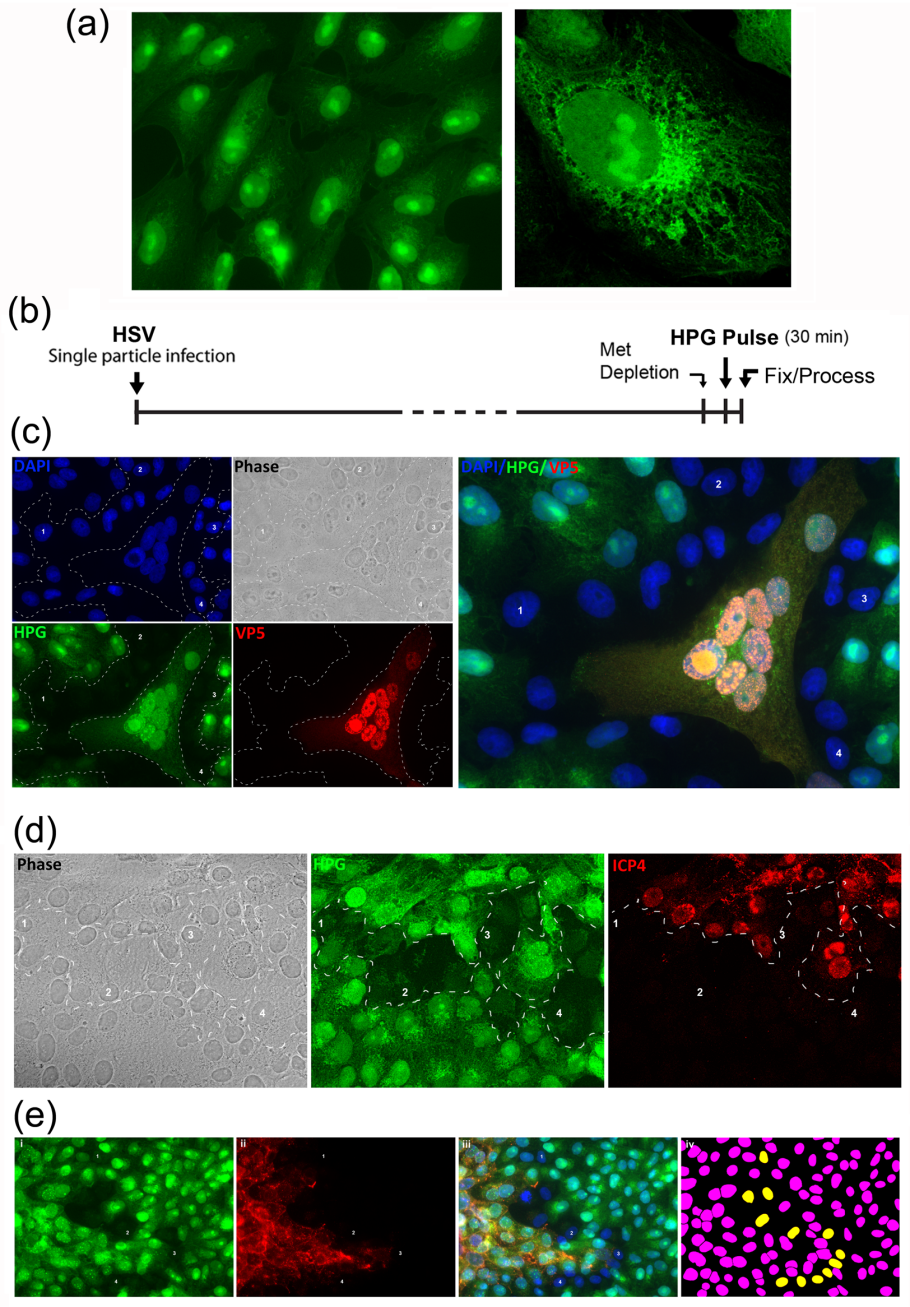
Spatiotemporal resolution of suppression and recovery of protein synthesis at single cell level during cell-cell transmission. (A) Representation of a field of uninfected cells pulse-labeled using 0.5 mM of HPG for 30 min, showing homogenous levels of protein synthesis in all cells. A magnified view of a single cell is shown in the right hand panel. (B) Workflow of standard HPG pulse-labeling during single particle infection. Cells were infected with single particle and pulse-labeled with HPG from 24.5 to 25 hr p.i. before processing. (C) Cells were infected with HSV-2[186] according to the standard workflow, and stained for VP5 (red), followed by the click reaction. Three different zones of protein synthesis (green channel) are demarcated by the dotted lines. The central zone of active protein synthesis corresponds to the developing plaque and is co-stained with a viral antigen marker (red channel). This zone is surrounded by cells (see DAPI staining blue channel) which are severely suppressed for protein synthesis but appear morphologically normal and with no apparent virus antigen yet. Reference cells are numbered in this zone and corresponding panels for DAPI and phase. This translationally supressed zone is further surrounded by cells exhibiting normal levels of protein synthesis. The merged image is shown in the right hand panel. (D) Cells were infected as above but with HSV-1[KOS] and stained for ICP4 (red), followed by the click reaction. (E) Quantitative analysis of protein synthesis levels based on HPG intensity (green) using Image Pro Plus software (Media Cybernetics). Translation levels were colour coded with yellow representing a threshold of 30% or below of the maximum observed and pink above this threshold (panel iv).

Typical results showing spatial analysis of a field of uninfected cells pulse-labeled with HPG for 30 min is shown in Fig 1a, with an individual cell shown at higher magnification in the right-hand panel. Pronounced localisation of newly translated proteins to the nucleus and nucleolus together with distribution throughout cytoplasmic organelles reflects previous observations by ourselves and others [34, 42–44, 47]. Overall incorporation levels within individual cells were relatively homogeneous.

The standard protocol for analysis of translation during HSV cell-to-cell spread is shown in Fig 1b. Confluent cell monolayers were infected at extremely low multiplicity (approximately 1 in 4000 cells infected), and infection allowed to proceed after addition of neutralising antibody to prevent secondary infection from free virus. After approximately 20–24 hr, cultures were pulse-labeled with HPG for 30 min and analysed by simultaneous click chemistry and immunofluorescence. Multiple early plaques were then inspected with each showing identical features regarding the main outcomes described below. Advancing infections were imaged with x63 or x40 objectives encompassing approximately 40 or 120 cells respectively on each field and at least 10 fields were evaluated. Each panel of a figure is representative of these fields. Typical images, analysing active protein synthesis in relation to virus spread (in this case with HSV-2) are shown in Fig 1c. The extent of infection is marked in this case by antibody to the late capsid protein VP5 (red channel). Total cell nuclei (DAPI staining) are shown in the blue channel and active protein synthesis during the labeling interval is shown in the green channel. The merged channels are shown in the right hand panel. Various zones discussed in the text are delineated by white dotted lines. A subset of individual cells are labeled for spatial reference. The extent of the focus of infection can therefore be seen, with typical abundant nuclear accumulation of VP5 together with efficient protein synthesis in those cells (HPG, green channel). Strikingly, immediately surrounding this area of infection there is an extensive area of cells, almost completely surrounding the central focus, where protein synthesis has been suppressed to virtually background levels. Cells in this zone appear morphologically normal (phase channel) and as indicated are not yet synthesising detectable levels of virus protein, at least VP5 in this case. Cells more distant to this zone (i.e. external to reference cells 1–4) exhibit normal levels and distribution of protein synthesis that would be expected of uninfected cells.

VP5, while an abundant protein and a sensitive marker for infection, is nevertheless expressed from early to late times in infection. We repeated these experiments using the immediate-early (IE) protein ICP4 as a marker for very early infection and expression of viral proteins, also examining HSV-1 in this case (Fig 1d). Here infection is spreading from the top of the panel downward. Again a broad zone, representing almost the entire front of the advancing infection, exhibited pronounced suppression of protein synthesis (green channel, representative cells marked 1–4 within the dotted lines). Cells in the lower region of the panel, more distant from the advancing front, again exhibited normal levels of protein synthesis. Delineating cells on the basis of protein synthesis i.e. within the dotted lines, it can be seen that cells in the shutoff zone express either undetectable or very low levels of ICP4, while cells at the top region exhibit much more abundant ICP4 and importantly overall protein synthesis levels have recovered to virtually normal.

A final example of this translational shutoff at the extreme front of an advancing infection, together with recovery in the immediately adjacent cells in shown in Fig 1e, in this case with infection marked by a glycoprotein gB. While the conclusions were clear from visual inspection, to spatially delineate the translational shutoff area in a more quantitative manner, we used the DAPI signal to create a mask for individual cell nuclei and then quantitated the protein synthesis signal within individual cells of the entire field, normalised for nuclear area. We set a threshold for significant shutoff at 30% or below the maximum for a field (i.e. an approximately 3-fold reduction, colour coded yellow). All other cells were coded pink. This method is somewhat conservative since translation levels in uninfected cells (i.e. in mock-infected monolayers) were relatively consistent, rarely exhibiting levels below 50–60% of the maximum in the field. Setting the threshold at 30% may miss cells that were in partial shutoff but this does not materially alter our conclusions. Thus, individual cells outside the shutoff zone (right hand side of the field) exhibited relatively limited variation. Cells within the shutoff could be clearly observed (coded yellow) with cells interior to this, and now gB positive, exhibiting restored levels of protein synthesis at approximately similar levels to those on the other side of the shutoff zone at the right hand side of the field.

Cells at the extreme front of a spreading plaque must be either uninfected or at the very earliest stages of infection. Cells that are progressively located inward from the boundary are generally at a later stage of infection. The simplest interpretation of these results therefore is that there is a biphasic suppression and recovery of protein synthesis. It is not likely that cells in the shutoff zone are uninfected. Rather cells at the leading edge, exhibiting efficient suppression of global translation over a broad spatial front, are likely at the very earliest stages of infection where ICP4 expression is not yet detectable, or is at very low levels. Importantly, cells immediately adjacent on the inward side have recovered from this shutoff to restore comparatively normal levels of total protein synthesis and progressively increasing accumulation of steady state ICP4, while cells immediately adjacent on the outward side represent cells which are not yet infected. Although it is presently impossible to precisely analyse it, considering the extent of infection and spatial distribution of normal levels of synthesis immediately adjacent to cells with almost total suppression, the biphasic suppression and recovery is likely to occur over a very short period of time (see below).

**Fig 2:**
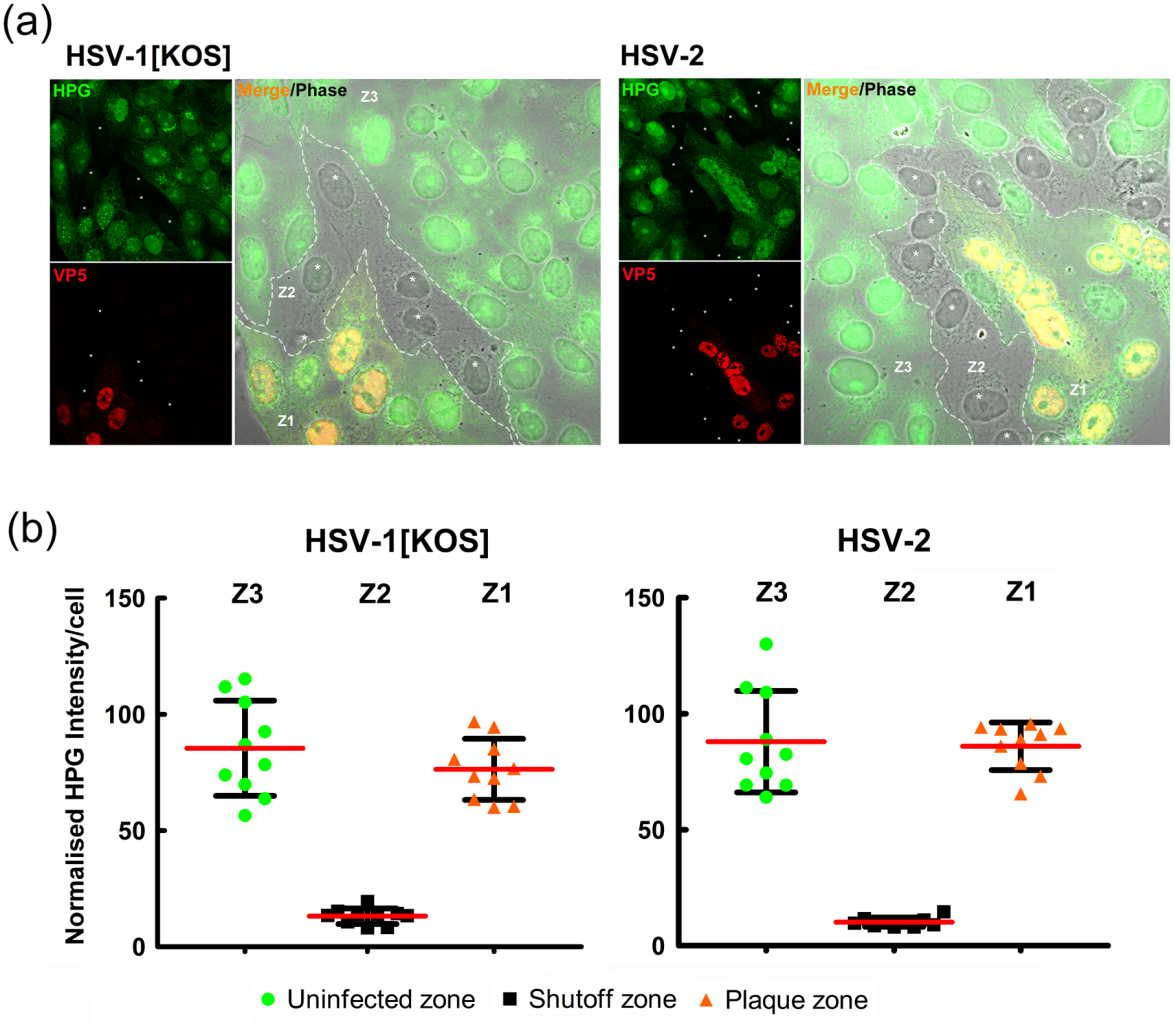
Comparison of translational suppression by HSV-1 and HSV-2 during cell-cell spread. (A) Cells were infected (MOI 10) with HSV-1[KOS] or HSV-2[186] according to the standard workflow in Fig 1b, analysed for newly synthesised protein (green) or VP5 accumulation (red). The merged panel also includes the corresponding phase images of the same fields. (B) Quantitative analysis of translation at the single cell level based on HPG intensity (green) in zones demarcated by the translational shutoff. Green dots indicate uninfected cells beyond the shutoff zone, black squares indicate those cell within a contiguous zone exhibiting shutoff and orange triangles indicate more central cells within the plaque, positive for VP5 and translational recovery.

To examine the degree of translational shut off at a single cell level in a more quantitative manner, we repeated our standard analysis, additionally directly comparing HSV-1 and HSV-2 (Fig 2). The results are quantitated for individual cells within zones delineated on the basis of a) translational levels, b) VP5 levels and c) spatial relationship. The selective regional suppression was again extremely efficient (Fig 2a). Almost every cell in the shutoff zone, zone 2 (Z2, in merged panel), directly adjacent to the advancing infected zone (Z1), exhibited very substantial shutoff of translation. Translation within cells in the advancing focus itself (Z1), even those cells immediately adjacent to the shutoff zone, showed virtually normal levels of overall protein synthesis, comparable to uninfected distal cells in Z3, i.e. those exterior to Z2. The quantitative analysis showed that while there was some cell-to-cell variability in the uninfected zone, this was modest and few if any cells were more than 1.5–2 fold different from the mean. By contrast translation in cells in the shutoff zone for HSV-1 were reduced to 5–15% of the mean of those in the uninfected zone, and there was a more uniform and almost complete shutoff for HSV-2. Importantly cells within the infected focus Z1, had returned to absolute levels only marginally lower than those in the uninfected zone.

Generally large areas of an advancing infection exhibited very efficient regional shutoff although this did not always result in a complete annulus of shutoff around the infection (likely due to inherent asynchrony in phasing, see below and Discussion), Nevertheless such a spatial distribution with an extended ring of translational shutoff could frequently be observed (see e.g. Fig 1c, S2a Fig)

### Cell type dependency and temporal modulation of the efficiency of shutoff

We examined spatial features of translational shutoff in several other cell types with generally similar results but noted one distinctive feature (S2 Fig). As indicated above, in Vero cells at 24 hr post infection (p.i.), translational shutoff at the advancing edge of virus spread was efficient, in this case forming a distinct and almost complete annulus around the advancing focus (S2a Fig Vero, asterisked cells). By contrast in parallel in human skin keratinocytes (HaCaT cells), while plaque formation was clearly advancing, shut off was quite difficult to discern, with no clear distinction at the periphery of the focus versus more distant uninfected cells at the perimeter of the field (S2 Fig HaCaT). However, when the pulse was delayed until later in the advancing keratinocyte infection, although general incorporation in uninfected distal cells remained similar, pronounced shutoff was now clearly discernible in cells immediately surrounding the now expanded focus of infection (S2b Fig, HaCaT 50 hr p.i.).

While there are several possible explanations for these observations, including paracrine effects progressively influencing distant uninfected cells (see Discussion), these results may reflect distinct phases of temporal modulation of translational control during infected cell to uninfected cell transmission, a process that would not be possible to observe with single-step population analysis.

### Translational suppression and recovery during single-step replication

**Fig 3:**
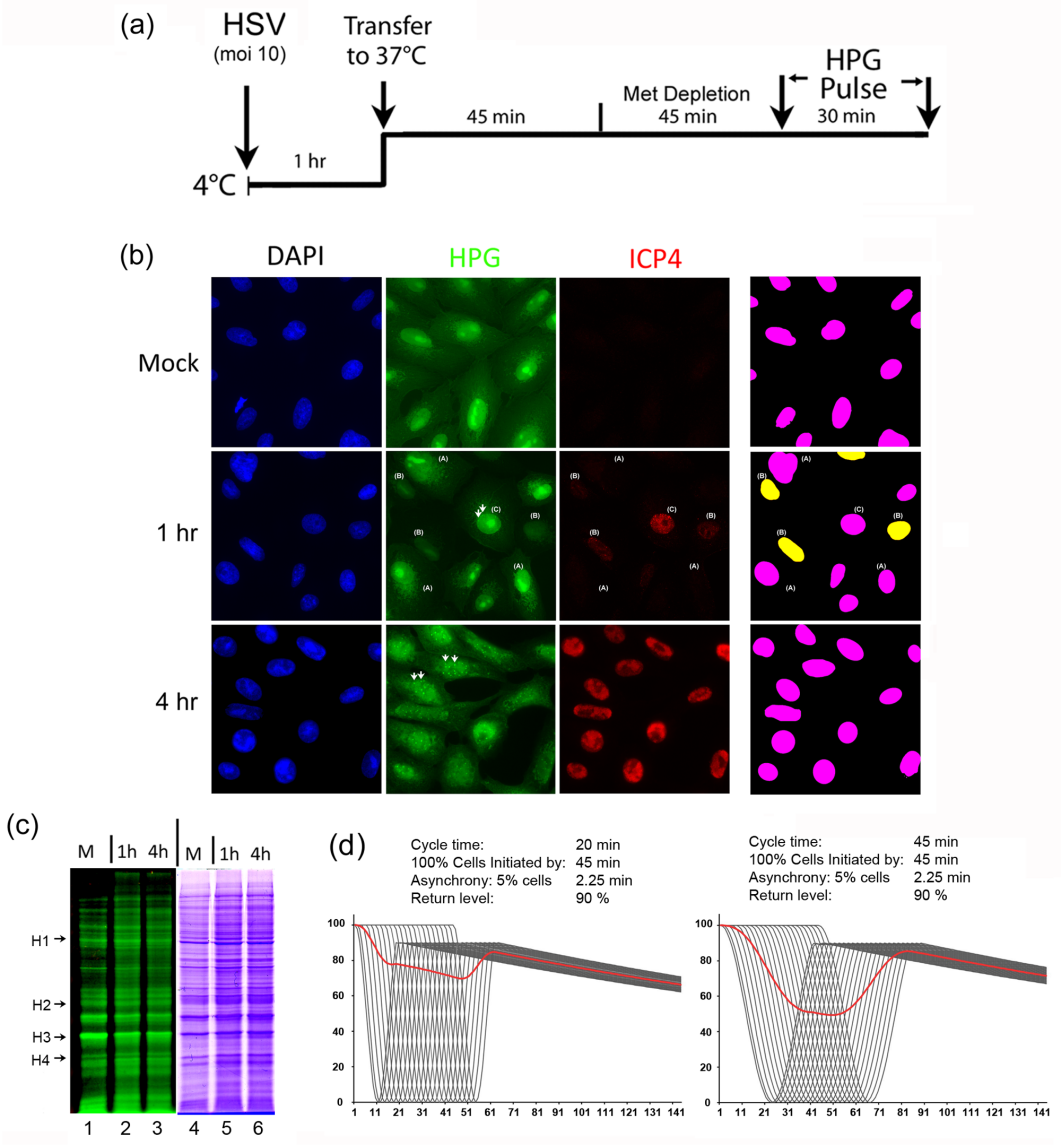
Biphasic switch of suppression and recovery during single-step replication. (A) Workflow of HPG pulse-labeling for the earliest analysis of translation during single-step replication. (B) Cells were synchronously infected (MOI 10) with HSV-1[KOS] and analysed for newly synthesised proteins and steady state accumulated ICP4 (red) at either 1 hr p.i. or 4 hr p.i. Cell classes with respect to protein synthesis levels are labeled A, B, or C as discussed in the text. NPD formation (arrowheads) and increased ICP4 levels are shown in a class C cell. Translation levels were colour coded with yellow representing cells reduced to 30% or below the maximum observed and pink representing cells above this level. (C) Vero cells were mock-infected or infected as above with the indicated strains of HSV and labeled either at 1 hr p.i. or 4 hr p.i. for 30 min. Cells were analysed as described for S1 Fig with newly synthesised protein profiles in the left hand panel and total protein profile of the identical gel in the right hand panel. (D) Simulation of an early oscillation in protein synthesis (though simulation applies to any output), with time after infection. The Y-axis represents levels of protein synthesis (% of uninfected) and the x-axis represents time (min). The model shows a hypothetical outcome where protein synthesis declines to zero and returns to a definable percentage of starting level (90% in this example), within a cycle time that can be also be set (30 min in the left hand panel). Thereafter protein synthesis declines at an exponential rate with a t_1/2_ that can also be set. However infection in a population is not instantaneous and it takes a finite amount of time for all cells to become infected. This is represented by an “asynchrony rate”, assumed to be linear for simplicity, and can also be varied in the simulation. Here all cells will be infected by 45 min at a rate of 5% of the population, every 2.25 min. (In principle, there could be a single curve for every cell in a population e.g. 10^6^ curves, but this is impractical for demonstration purposes). Each black curve therefore represents 5% of the population, there are 20 such curves and the oscillating cycle is initiated in all cells by 45 min. The outcome for each curve (i.e. each 5%) is shown, together with the total output, at any one time, averaged across the population (red line). Even where the output could be sample instantaneously, the averaged output declines by only a modest amount, and would not reflect the reality of a decline to zero in each cell. In the example in the right hand panel, the cycle time has been lengthened to 40 min with other parameters the same. Even here, the total, maximal decline is still less than two fold.

Overall translation declines progressively during HSV infection, with several mechanisms contributing to such decline including the vhs function (UL41) [35, 37, 49–57]. The kinetics and the extent depends on several factors including virus strain, with HSV-2 promoting a more rapid decline, consistent with our results above. Nevertheless, this translational decline is generally explained as a continuum within any one cell, of progressive unimodal early repression dependent upon vhs (among other factors) and continued later repression by independent mechanisms, summed across the population. Our conclusion from single cell analysis of a very rapid early but biphasic programming of suppression and recovery of overall translation is not accounted for in current models. In population based single-step experiments (S1 Fig, high MOI infection and SDS-PAGE), we did not observe significant early shutoff of translation. We repeated these experiments using high MOI infection and analysing protein translation in the same cultures, either by imaging analysis at the single cell level or by SDS-PAGE and in-gel fluorescence. The protocol (Fig 3a) was adopted after evaluating procedures to obtain as synchronised an infection as possible (MOI 10 with pre-incubation at 4°C), at the earliest time frame and with efficient labeling sensitivity as possible (see experimental procedures). Cells were processed and colour coded as for Fig 1. In mock-infected cells we observed the typical intracellular distribution of newly synthesised proteins and minor cell to cell variability (Fig 3b, mock, HPG and right hand panel). In contrast, in HSV infected cells, we observed distinct variation in translational levels at 1 hr after infection, with three classes of spatially interspersed cells (Fig 3b, HSV-1 1 hr). In class A cells (labeled A), there was little alteration in levels of protein synthesis (HPG and right hand panel) from those seen in mock-infected cells and either no, or barely discernible, ICP4 expression. In class B cells, there was a pronounced translational shutoff with such cells expressing low but clearly detectable levels of ICP4. In other cells (class C), overall protein synthesis levels were at least as high as those in mock-uninfected cells. In these cells, the qualitative patterns of localisation of newly synthesised proteins exhibited a combination of those seen in uninfected cells (including abundant nuclear and nucleolar accumulation) together with additional distinct features termed NPDs, which are hallmarks of advancing infection [34]. These cells showed increased levels of ICP4 accumulation compared to class B cells. Taking into account the results during HSV cell to cell spread after low MOI infection, we interpret these data as follows.

Class A cells represent cells that are either uninfected, or formally at the extremely early stages of infection, even prior to translational shutoff and prior to detectable ICP4 levels. Although at MOI 10 essentially all cells (99.995%) will be infected at some particle level, infection will show some degree of asynchrony and it is difficult to discriminate between these two possibilities. However, we think it is more likely that class A cells are infected but not yet exhibiting significant translational shutoff or ICP4 synthesis. Class B cells (approximately 25%) represent cells which are at some stage within the suppression/recovery cycle in protein synthesis, exhibiting much reduced levels of protein synthesis together with limited levels of ICP4. Class C cells are then further advanced, exhibiting increased levels of ICP4, but also increased levels of total protein synthesis compared to class B cells. Consistent with this, pulsing later in infection (4 hr) the vast majority of cells now exhibited totals levels of protein synthesis that were similar to uninfected cells, together with increased and more uniform levels of ICP4 (Fig 3b, 4 hr). As expected, these cells also showed the advancing features of NPD formation (white arrowheads) combined with abundant nuclear import [34] and early stages of replication compartment formation. However in absolute terms, the majority of total protein synthesis at this stage still represented host protein synthesis, as seen from the parallel analysis of the same cells at the same time point by SDS-PAGE and in-gel fluorescence (Fig 3c). Thus while at a single cell level, a significant fraction of the cells exhibited very substantially reduced levels of total protein synthesis at 1–1.5 hr, this was not evident from SDS-PAGE analysis of active protein synthesis and the considerable bulk of synthesis represented host proteins (exemplified by representative host species (Fig 3c, cf. lanes 1–3, host species labeled H1-4). Analysis of advancing cell-cell transmission therefore reveals a process, whereby efficient shutoff and restoration of translation takes place in a rapid manner that is not as readily experimentally assessed during spatial analysis of high MOI infection and or by biochemical analysis at the population level. We constructed a modelling simulation which accounts for this contrast (Fig 3d). The simulation, while necessarily oversimplified, allows variable inputs for several parameters including; overall time for all cells to become infected (e.g., 45 min in Fig 3d), rate of infection (degree of asynchrony, e.g. 5% of cells every 2.25 min), the overall translational cycle duration and the restoration level as a percentage of that in uninfected cells (see Fig 3d legend). This simulation which is within physiological expectation for a high MOI infection, shows that every cell in a population could be completely supressed to zero translational activity (black lines, each representing 5% of the population) and with a biphasic oscillation return to a definable high level in a certain time frame, yet this would not be discernible from the averaged population output at any time (red broad line). This represents a type of technical sampling limit well known in analysis of oscillating outputs, combined with averaged population analysis inherent in current biochemical techniques versus information gained from single cell analysis of metabolic processes (see Discussion).

### A single particle of HSV-2 can promote transient global translational suppression

**Fig 4:**
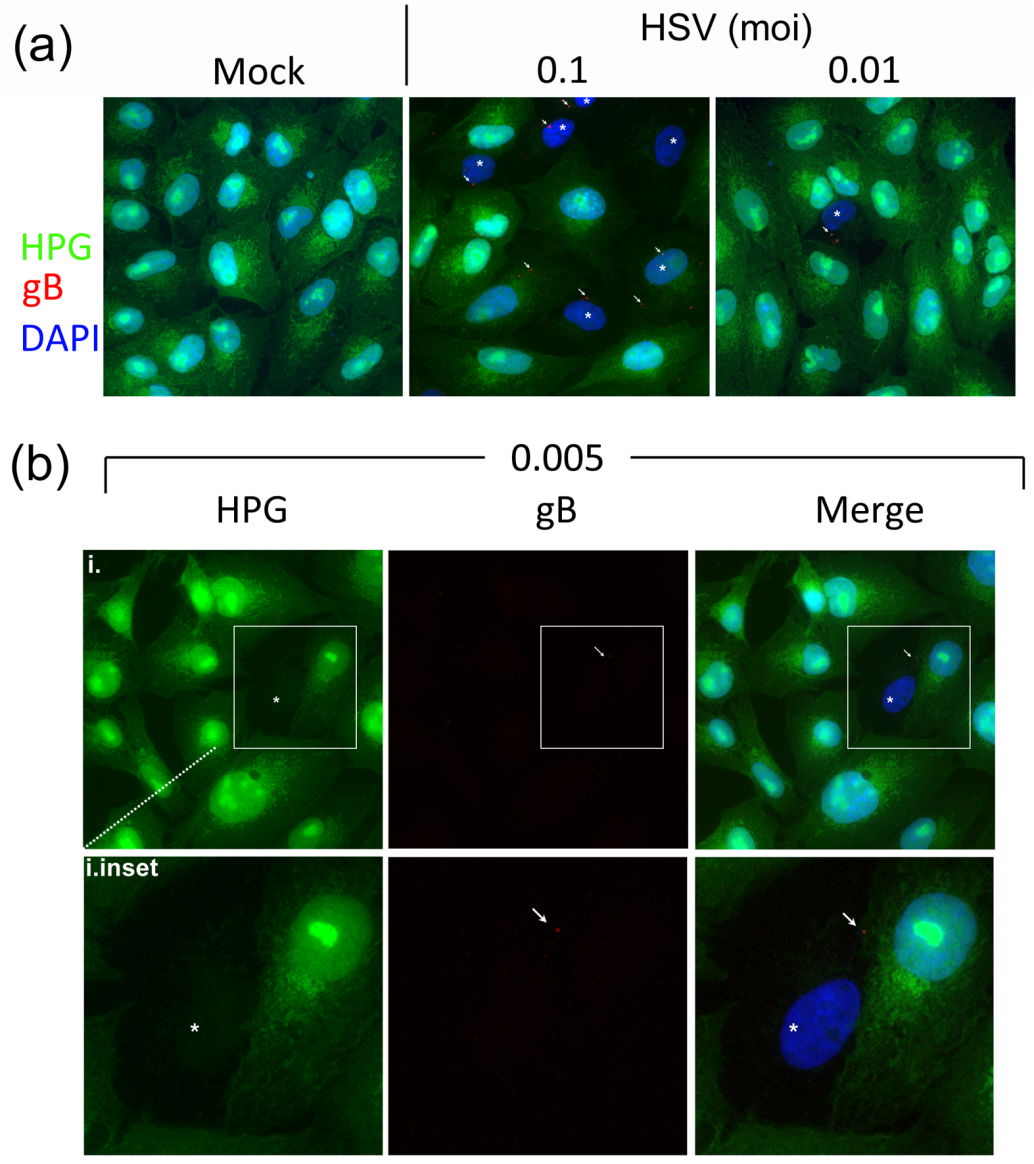
A single particle of HSV-2 can induce profound general translational suppression. (A) Cells were pulse-labeled for 30 min at 1 hr after mock-infection or infection with HSV-2[186] at MOIs of 0.1 or 0.01 as indicated, then fixed and analysed for newly synthesised proteins (green), gB (red) and total cells (DAPI). Asterisks denote the cells in shutoff phase, and diagonal arrows indicate gB. (B) Cells were infected with HSV-2[186] at MOI of 0.005, (i.e. 1 infectious pfu/200 cells) fixed and processed as standard. The insets for panels i and ii represent magnified views of individual cells. Asterisks denote the cells in shutoff phase, and diagonal arrows indicate gB, always observed as a focus.

Cell-to-cell transmission during plaque spread involves high numbers of particles transmitting to the surrounding uninfected cells. To examine whether a single particle would be sufficient to induce efficient global translational shutoff, we infected cells with progressively decreasing amounts of virus, down to a MOI 0.005 where 1 cell in 200 would be infected with an infectious particle and statistically the probability of infection by more than one particle is extremely low (<0.005%). We then pulse-labeled cultures with HPG 1 hr after infection. Under these conditions, detection of individual particles themselves was difficult but antibody to gB gave brighter signals than several other antibodies evaluated including ones to VP5. The results (Fig 4) showed that the numbers of cells showing translational shutoff titrated downwards in proportion to increasing virus dilution (Fig 4a). At MOI 0.01 isolated individual cells showing pronounced shutoff of translation were observed, a feature never seen in mock infected cultures (Fig 4a). Moreover such cells contained detectable fluorescent punctae, stained by the antibody to gB. At MOI 0.005, isolated individual cells could still be observed, with virtually complete translational shutoff, examples of which are shown (marked by asterisks, Fig 4b and inset). In these cells, defined by the virtual absence of detectable translation, corresponding single particles could be observed (marked by arrows, panels i inset). We note that although there have been few previous reports on the fate of membrane proteins very early after infection, translational shutoff indicates that these cells were infected, and thus the signal for gB, indicates that the membrane, or at least a significant population of this component remains in a tight localised focus. (As explained below, translational shutoff requiring simply virus attachment rather than infection would be not be consistent with any known mechanism nor with further results from this work). These results make no assumption about non-infectious particles and indeed it could be that certain particles we detect did not go on to make an infectious pfu, but were sufficient to promote shutoff. However even accounting for particle/pfu ratios, (in our stocks approximately 50), the vast majority of cells at MOI 0.005 would be infected by a single particle. While it may be, and indeed is likely, that particles go undetected in this experimental set up, nevertheless taken together our results strongly support the conclusion that at most a few particles and very likely a single particle of HSV-2 can be sufficient to induce a very profound translational shutoff. We note however that while HSV-1 exhibited prominent regional shutoff during cell-cell spread (Figs 1–3), we could not observe shutoff under conditions of single particle HSV-1 infection.

### HSV vhs promotes transient global translational suppression

**Fig 5:**
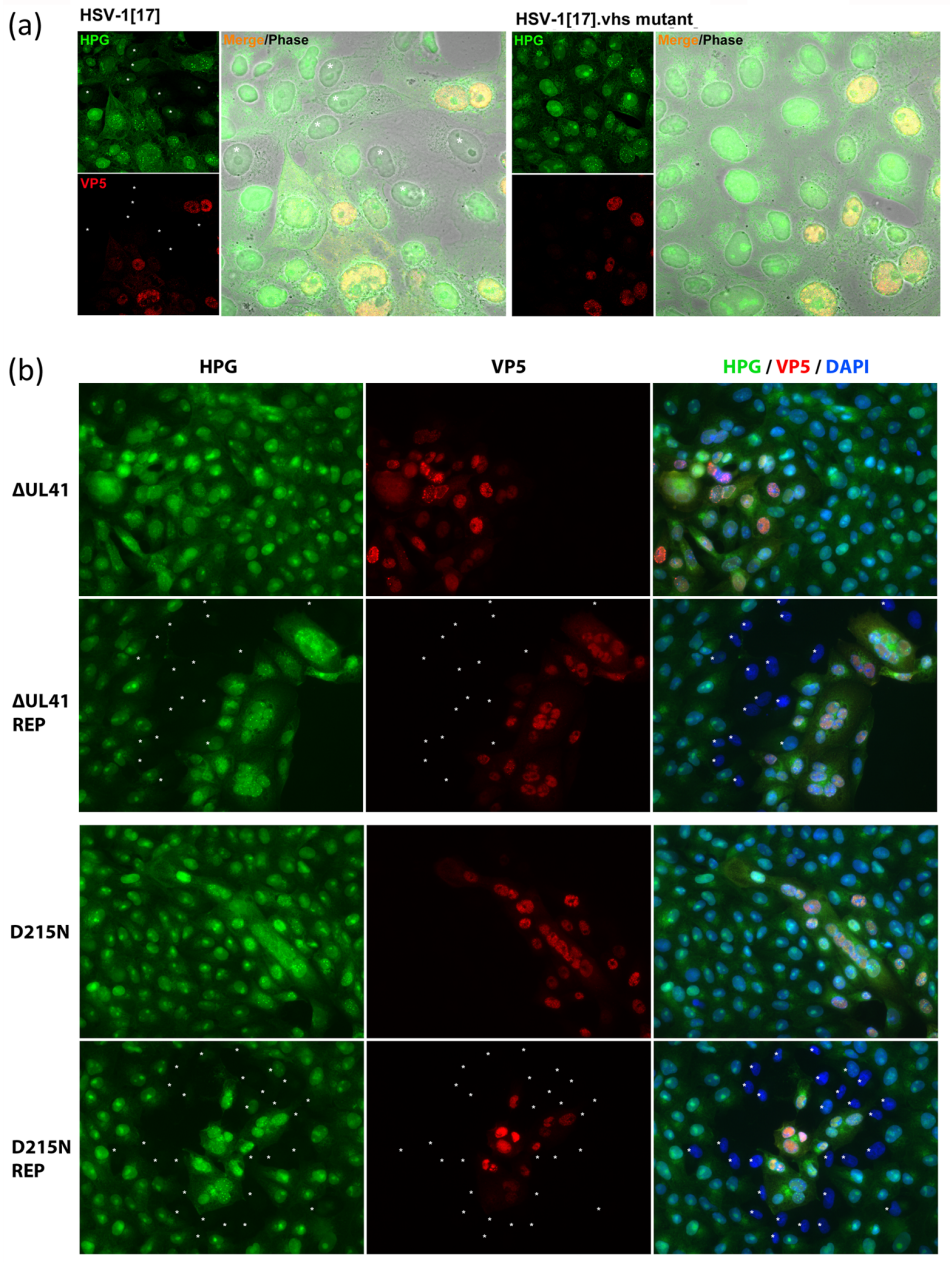
vhs accounts for the translational shutoff zone in an advancing infection. (A) Cells were infected with either HSV-1[17] or HSV-1[17].Δvhs according to the standard workflow in Fig 1b, and analysed (25 hr) for newly synthesised protein (green) or accumulated VP5 (red). White asterisks denote the cells in shutoff phase. (B) Cells were infected with different mutants of HSV-2[186]; ΔUL41 or its repaired strain ΔUL41-REP and D215N or its repaired strain D215N-REP and analysed as above (25 hr). White asterisks denote the cells in shutoff phase.

As indicated above, overall protein translation declines progressively during HSV infection, contributed to by multiple mechanisms including the HSV vhs function [14, 35–39]. To examine the influence of vhs on regional translational suppression at the single cell level, we examined a series of deletion or catalytic vhs mutants in HSV-1 and HSV-2, with representative results shown in Fig 5. While HSV-1 [17] exhibited efficient regional shutoff (Fig 5a, HPG asterisked cells surrounding VP5+ve cells), for HSV-1[17].Δvhs we never observed significantly declined translational shutoff at the plaque periphery. This lack of regional shutoff for a vhs mutant was even clearer with HSV-2, given the profound suppression for the parental virus. We first tested a complete deletion mutant HSV-2.ΔUL41 (ΔUL41), versus its repaired counterpart, ΔUL41-Rep. For the repaired virus, there was virtually complete cessation of translation in numerous cells immediately surrounding the advancing focus of infection (Fig 5b, ΔUL41-Rep, asterisks). For the mutant, no zone could be identified and there was little discernible difference in cells immediately adjacent to the infected focus versus more distant cells. To complete these analysis, we examined a HSV-2 vhs mutant containing a single amino acid substation, D215N, which inactivates the RNase function of the protein together with its repaired version [58]. The repaired virus again showed very pronounced regional shutoff, with virtually every cell surrounding the focus of infection being suppressed while cells within the focus showed overall levels of translation similar to those more distant peripheral cells (Fig 5b, D125N-Rep). By contrast, again we never observed significantly altered levels of protein synthesis in cells surrounding the focus of D215N mutant infection (Fig 5b, D215N). Since in the D215N mutant, vhs is packaged into the virion in similar copy numbers to the repaired mutant [58], these results indicate that the translational shutoff is tightly linked to the RNase activity of vhs and that infection and tegument deposition is necessary for shutoff. The results also imply that, while other mechanisms play a role in host translational shutoff, in this regional shutoff at the earliest stages of infection, vhs plays a critical role.

### Relocalisation of eIF4H coupled to suppression of protein translation during HSV progression

**Fig 6:**
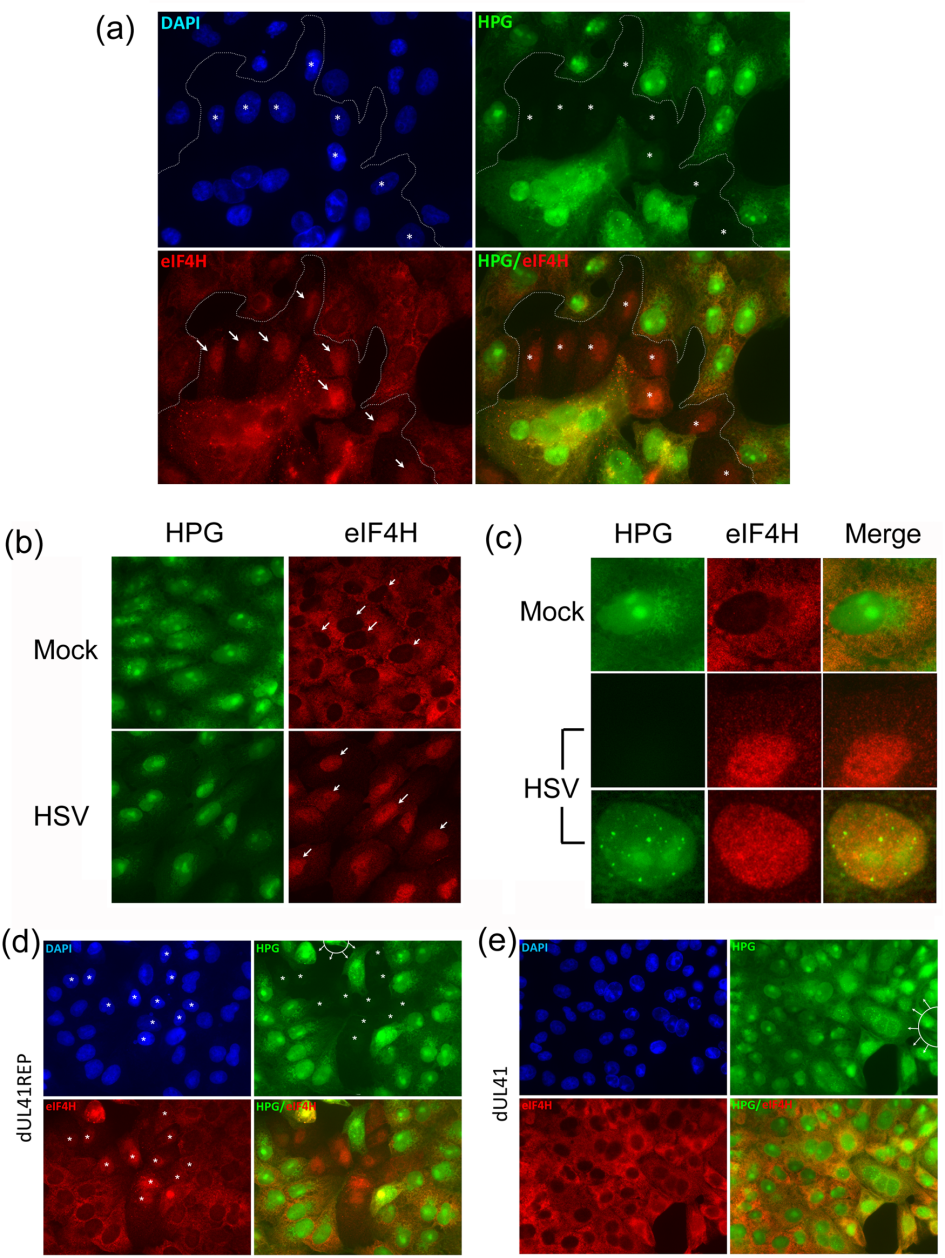
eIF4H nuclear relocalisation is coupled to the suppression of global protein synthesis. (A) Cells were infected with HSV-2[186] according to the standard workflow and analysed (25 hr) for newly synthesised protein (green channel) or eIF4H localisation (red channel). Uninfected cells are at the topmost region of the panel with infection emanating from bottom left (as reflected by diagnostic NPD formation). White asterisks (DAPI, green channels) denote the cells in shutoff zone, demarcated by the dotted line. Diagonal arrows denote the same cells showing relocalisation of eIF4H to the nucleus in the shutoff zone while cells in the topmost region show cytoplasmic localisation. (B) For single step analysis, cells were synchronously infected with HSV-1[KOS] at MOI 10 and labeled at 2 hr p.i. for newly synthesised proteins (green) and stained in parallel for eIF4H (red). Diagonal arrows (in eIF4H panel) indicate the nuclei for mock-infected cells and infected cells respectively and the dramatic shift in relative eIF4H localisation in infected cells which are now actively synthesising proteins and exhibit NPD formation. (C) Magnified view of individual cells showing a mock-infected cell with typical eIF4H cytoplasmic localisation compared to high MOI infection showing cells in the shutoff phase (2^nd^ row) or recovery phase with restored protein synthesis, nucleolar import, NPD formation (3^rd^ row). eIF4H is relocalised to the nucleus during shutoff and retained there during translational recovery. (D, E) Infection as standard for localisation after low MOI infection and transmission (as in Fig 1b). Monolayers were infected with 50 pfu of ΔUL41-REP (D) or its mutant strain ΔUL41 (E) and labeled for protein synthesis (25 hr p.i.). Cells were then analysed for total protein synthesis and eIF4H localisation. The focal origin of infection is denoted in the green channel by a white semicircle with emanating arrows. For ΔUL41-REP, white asterisks (DAPI, green channels) denote cells in the shutoff zone, exhibiting pronounced translational suppression. These same cells are indicated for eIF4H localization (red channel). Asterisks are omitted in the merged channel. For the mutant ΔUL41, protein synthesis levels and localisation were observed together with progressive NPD formation, no shutoff zone was observed nor was there any relocalisation of eIF4H at the boundary of advancing infection.

These data link the regional oscillation in translational levels to vhs function. We next wished to examine any corresponding regional alteration of factors within the translational apparatus related to vhs function. In vivo, vhs is thought to promote the degradation of mRNAs that are in the process of translational initiation by interaction with candidate cell translation factors, notably eIF4A, an RNA helicase and eIF4H, an accessory factor that stimulates eIF4A [38, 41, 59, 60]. We therefore simultaneously analysed protein synthesis by HPG incorporation and steady state levels and localisation of a series of translation factors during HSV cell-cell spread. S3 Fig shows representative images for eIF2α, eIF4AII, eIF4B, and eIF4G at the leading edge of virus spread, containing examples of individual cells with significant shutoff (cells marked 1) adjacent to external cells with normal levels of translation (cells marked 2). We could discern no difference in levels or localisation of any of these components in cells where translation was suppressed compared to adjacent cells showing normal translation (S3 Fig). However we observed a very striking and clear cut specific relationship between translation and localisation for another factor, eIF4H (Fig 6). In this case, virus spread is advancing from the bottom left with cells at the top being uninfected. A profound shutoff zone is seen across virtually the entire advancing face (Fig 6a, HPG and DAPI, cells marked with asterisks). While eIF4H is localised predominantly within the cytoplasm in cells in the unaffected top zone, there was a pronounced and highly correlated relocalisation of eIF4H from cytoplasm to the nucleus in cells precisely within the shutoff zone (Fig 6a, HPG versus eIF4H, asterisks and arrows mark the same cells for each read out). Interestingly, eIF4H localisation in cells within the interior infected zone also appeared more prominently nuclear. To extend this, we also examined eIF4H localisation early after infection at high MOI at a time (2 hr) when all cells would be expected to be infected (but the asynchronous pattern of shutoff and recovery would be expected within individual cells across the population, as described in Fig 3). The results showed a clear and pronounced relocation from the mainly cytosolic localisation in mock-infected cells to a distinctly nuclear localisation in many HSV-infected cells (Fig 6b, arrows indicate eIF4H localisation in representative cells for mock and infected). An important feature of these results in shown in Fig 6c. This shows the typical pattern of protein synthesis levels and localisation in mock-infected cells, alongside the mainly cytoplasmic eIF4H distribution. In HSV infected cultures we detected two patterns. A representative cell showing almost complete shutoff is shown together with pronounced eIF4H translocation into the nucleus. However infected cells exhibiting translational recovery, combined with progressive NPD formation, clearly show the retention of eIF4H in the nucleus notwithstanding translational recovery. The significance of these results for shutoff and translational mechanism are discussed further below.

Finally considering the link with vhs function, we wished to determine whether eIF4H relocation was dependent upon vhs activity. We therefore compared regional translational activity and eIF4H localisation for the vhs deletion (ΔUL41) and repaired virus (ΔUL41-Rep). Consistent with the data above, for ΔUL41-Rep, we observed a pronounced zone of efficient translational suppression (Fig 6d, zone marked by asterisks, HPG and DAPI) surrounding the advancing infected focus (identified by phase microscopy and indicated by top semicircle), with a clear correlation between translational shutoff and eIF4H relocalisation to the nucleus (Fig 6d, HPG and eIF4H, asterisks). Both the regional shutoff and eIF4H relocalisation were abolished in ΔUL41. We never observed either process for the mutant with a typical field shown in Fig 6e.

## Discussion

### A bimodal temporal switch in protein translational

**Fig 7:**
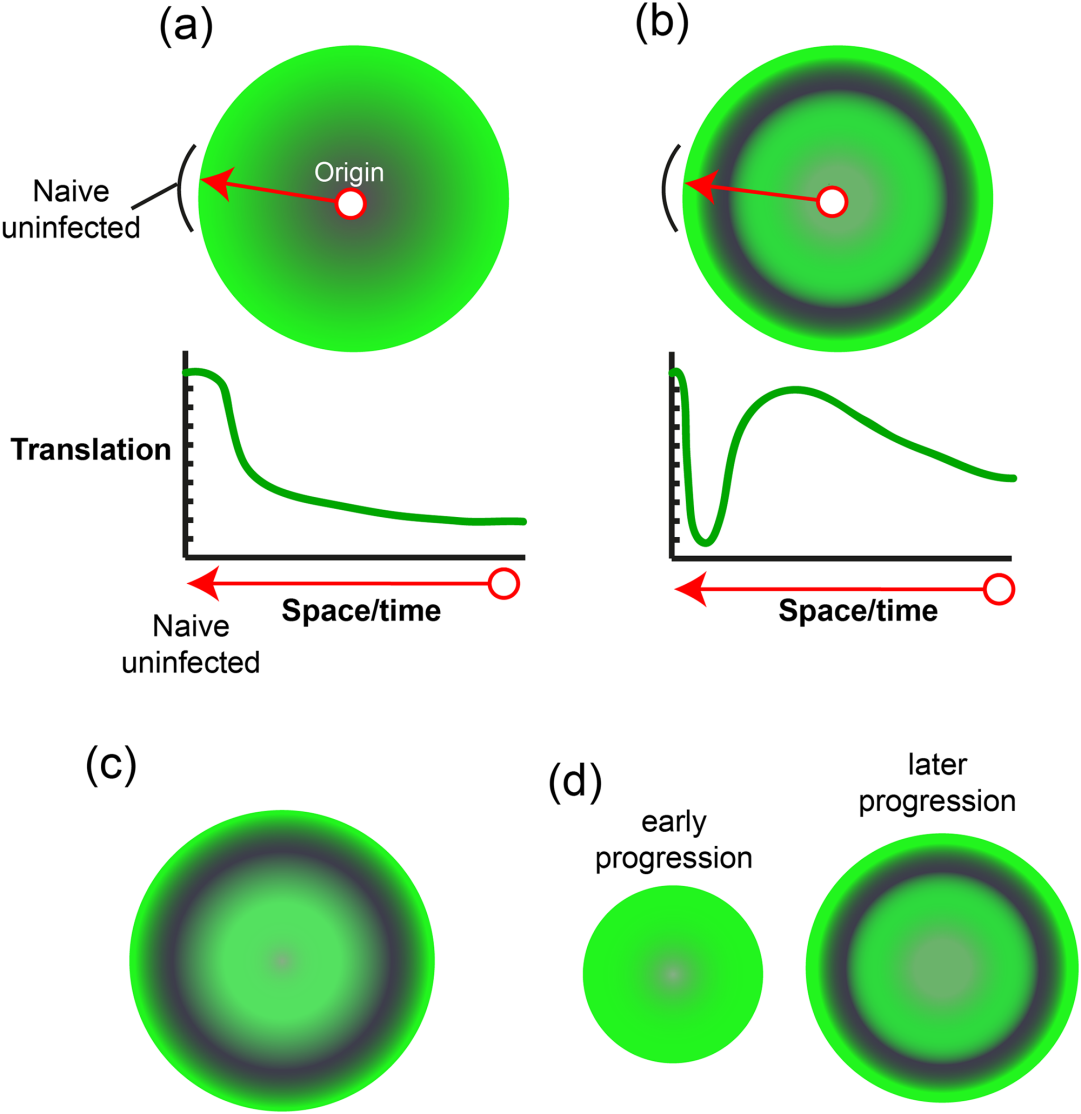
Schematic representation of spatial translational activity during plaque progression. (A) Current view anticipated from present data. Infection initiating at the focal centre of infection (red circle) radiates outward (red arrow), with naïve uninfected cells at the extreme perimeter. Immediately adjacent internal cells represent the earliest/most recently infected cells and progressively more central cells represent the progressively later/“older” infected cells. Absolute translation levels are indicated in green with declining levels indicated by increasing grey. A cross-sectional slice (arrowed line) indicating relative overall levels from peripheral to central cells, is summarised in the graph below, where the x-axis indicates space from external to internal and therefore time from new to old. (B) A revised model shows distinct regionalisation of overall translational activity, with early pronounced suppression at the periphery together with restoration of translational levels in the more interior cells. (C) Model of an extended regional shutoff zone due to translational suppression in an increased numbers of cells as discussed in the text. (D) Spatial representation of translational activity in HaCaT cells during early progression (left scheme), where no apparent advanced shutoff zone is observed and later progression (right scheme) where uninfected naïve cells become progressively more susceptible and advanced shutoff operates.

From many previous studies, it is generally understood that HSV infection leads to increased, temporally regulated virus protein synthesis combined with a suppression of cellular protein synthesis, with overall translational rates declining from those seen in uninfected cells [35–37, 39, 52, 53, 57, 61–64]. This general decline in global cellular translation is proposed to occur in distinct phases reflecting firstly a vhs-dependent early shut-down (associated with the vhs RNase activity and potentially other vhs activities), transitioning into a general vhs-independent decline in translation, through multiple specific and non-specific pleotropic effects of infection (for reviews see [52, 65]). Our understanding of these processes has been gained from studies of cell populations (or biochemical analysis of population extracts) during single-step replication. The principle conclusions when applied to a multistep system, encompassing infected to uninfected cell transmission that occurs in a natural infection, would result in a spatial representation of translational activity as summarised in the schematic (Fig 7a). Thus infection initiating at the focal centre of infection (red circle) radiates outward (arrow), with naïve uninfected cells at the extreme perimeter. Immediately adjacent internal cells represent the earliest/most recently infected cells and progressively more central cells represent the progressively later/“older” infected cells. Absolute translation levels are indicated in green with declining levels indicated by increasing grey. A cross sectional slice (arrowed line) indicating relative overall levels from peripheral to central cells, is summarised in the graph below, where the x-axis indicates space from external to internal and therefore time from new to old. Though idealised, this represents a first approximation of what would be anticipated from current models but this is not what is observed. Instead we observed a distinct regionalisation of overall translational activity, with early pronounced suppression at the periphery together with restoration of translational levels in the more interior cells (Fig 7b and lower graphic). While overall translation is certainly reduced in the inner-most/older cells, this is not as extensive as anticipated.

This advancing zone of translational oscillation has important implications. While other factors and viral genes could play some role (e.g. activation and counter-activation of PKR mediated translational repression or stress mediated ATF6/PERK signalling), [15, 66–71], it is clear that the main mechanism of initial suppression (though not necessarily translational recovery) is viral mediated rather than cell mediated. This conclusion stems from the observation that vhs is critical and without vhs activity relatively little suppression of translation was observed, even at the single cell level. Because host-promoted suppression of translation can be counteracted by virus factors [11–15], it could be anticipated that in the absence of vhs, these measures and countermeasures could balance out. However the relative contributions of each of these processes have not been definitively dissected. Moreover, a main point of this current study is that such dissection by population-based studies would in any case be extremely difficult. As described above, population based averaged output may not have the resolution to discriminate distinct processes and factors influencing translation activity in space and time (e.g. Fig 3d and accompanying text). In future studies, it will be interesting to examine translation at the single cell level, (in both single-step and cell-cell transmission models), using single or double viral mutants where vhs is inactivated with or without other viral genes such as ICP34.5 or US11, thus blocking virus-induced translational suppression and virus-encoded counter-suppression that might have different contributions at different times.

In this regard it is interesting to consider what factors would potentially extend the regional shutoff zone, with the result simulated in Fig 7c. By this, we mean not simply more pronounced translational suppression in a given target cell population, but increasing the numbers of cells in a broader regionally defined zone, shifting the balance of the efficiency, or duration of shutoff versus the recovery in translation. These outcomes would be dictated by multiple factors, potentially with changing relative contributions at different stages of infection. Moreover, although vhs plays a key role in the regional shutoff at the earliest stages of infection, other factors or gene products which affect translational activity generally (or in a gene specific manner), could impact the overall spatial stratification. Examples include, increased half-life of input vhs, increased specific activity of vhs (as has been speculated upon previously [72], advanced de novo expression of new vhs activity, decreased activity or expression of virus factors that negatively modulate input vhs, such as VP16 and VP22 [72–76], and decreased capacity of the host to restore translation levels. Linked to this therefore is the question of the mechanism(s) involved in translational recovery, both with regard to host and viral functions. At its simplest, translational recovery could be explained by progressive loss of input vhs protein and relatively unaltered cellular translation machinery, without a requirement for specific vhs dampening and/or a translational restoration processes.

However clearly other factors are likely to play a role. With regard to the role of transcription in translational recovery, while it could be proposed that that abundant de novo host transcription was required, it could also be that translational suppression involved only a specific subpopulation of mRNAs (e.g those at some stage of the active translation cycle), and that translational recovery could ensue with mRNAs already made but in transport or not yet in active translation (see also below).

In addressing the question of whether any de novo virus gene expression is required, or at least promotes translational recovery, clearly this cannot be approached using cycloheximide or other translational inhibitors (to block de novo virus protein synthesis), since this would inhibit the activity to be measured. Blocking transcription, at a delayed time point after allowing some virus spread, and then pulsing with HPG to examine regional shutoff could in principle help address the question. The logic would be that, if the timing was appropriate, blocking de novo transcription in a cell which had received vhs and exhibited translational shutoff, (but had not yet restored normal levels), might result in prolonged shutoff within cells, thus extending the zone and numbers of cells suppressed. However such attempts did not yield interpretable results (in both single step and multi-step replication models). Although after high multiplicity infections vhs can clearly degrade host mRNA in the presence of actinomycin D, in attempts to examine overall spatial translational activity, we found that protein synthesis in control cultures was affected by actinomycin D even early after application and the dynamic range of the assays was severely reduced. A more fruitful approach for future studies will be to examine the extent of regional translational activity at the single cell level during infection with mutants, especially in components such as ICP34.5, VP16, or VP22 which have been reported to directly interact with vhs and to suppress its activity later in infection.

One additional aspect of note stems from the comparative analysis of human keratinocytes, i.e. a cell type that is perhaps more physiologically relevant for HSV infection. Thus, at times when in Vero cells broad regional shutoff in the advancing zone was readily discerned, this was not apparent in the keratinocyte model, even for infection with HSV-2. Overall protein synthesis measures, i.e. levels and general localisation, were approximately equivalent in HaCaT and Vero. However, as infection progressed in keratinocytes, distinct shutoff zones in advance of the antigen positive cells became much more apparent. This result is illustrated schematically in Fig 7d and warrants speculation on possible explanations. It could be that in keratinocytes, the numbers of virus particles transmitting from cell to cell early in infection is insufficient to induced translational shutoff. Then as infection progressed, whether through increased virus yields per cell or increased transmission rates to susceptible cells, shutoff was more efficiently induced because more particles infected the cells. This is possible but considering that a single particle of vhs, at least of HSV-2, could elicit shutoff in Vero cells, this explanation would require also that HaCaT cells were intrinsically more resistant to vhs-mediated shutoff, (although later non-specific translational suppression might still occur), a proposal that will be assayed in the future analysis using the single particle assay. Cell-type differences in the robustness or resilience of the translational apparatus (for example the availability of translation factors) has been previously alluded to, including the prospect that such intrinsic differences could influence the activity or requirement for vhs [77]. Such cell-type differences could help explain the observations reported here. However it could also be, at least in certain cell types, that quite distinct processes influence the outcome of infection when uninfected, naïve cells are in contact with or exposed to infected cells. For example, it could be in certain cells that paracrine mediators from infected cells signal to uninfected cells, promoting their relative resistance to translational shutoff and that as infection progressed these paracrine processes waned, or were counteracted by the virus, resulting in more efficient shutdown later in the progressing infection. Whatever the precise explanation, these results would inherently not be obtained in a model system of population analysis of single–step infection, and reinforce the utility of single cell analysis in transmission models in revealing additional complexity in virus-host interactions.

### Global translational suppression by a single HSV-2 particle

Our results strongly indicate that a single particle of HSV can be sufficient to promote translational shutoff. This stems from the considerations; 1) at the extremely low input virus used, the statistical probability that more than one particle was infecting the cells is extremely small, 2) we could observe the single particles responsible and 3) under the same conditions HSV-1 was unable to promote shutoff. Although in these experiments it is possible that some particles may have escaped detection and formally that some cells may be infected by more than one particle, taken together our results strongly indicate that a single particle of HSV-2 can suffice to promote transient, global shutdown. Unfortunately no useful antibodies are available for immunofluorescence analysis of virion associated input vhs, (or even de novo synthesised vhs) to directly assess vhs distribution in relation to shutoff and analysis of input vhs by immunofluorescence has never to our knowledge been reported. It is estimated that vhs is a relatively minor virion component with approximately 200 molecules incorporated per particle on average [51, 62, 78, 79] and our results indicate that the catalytic activity of these molecules is sufficient to promote pronounced, reversible translation shutdown. It will be interesting in future work to combine the HPG pulse labeling approach with single molecule RNA in situ hybridisation to host mRNAs, to examine questions on the abundance, location, selectivity and possibility temporal reversibility of RNA molecule abundance in relation to spatial outputs of active translation. We are currently attempting to combine bioorthogonal labeling and click chemistry with simultaneous RNA FISH to address such questions. In principle, the activity at the single particle level of HSV-2 versus HSV-1 could be due to a number of factors, including increased vhs copy number in HSV-2, increased specific activity (or half-life) of HSV-2 vhs, or less efficient/slower reversal of vhs activity, for example by association with de novo expressed VP16 or VP22. While formally each of these could contribute, it has been shown both in the context of virus infection and in reconstruction assays measuring activity of the individual protein, that HSV-2 vhs is more potent than HSV-1 [80–82]. The simplest explanation therefore to account for the observation that a single particle (or low numbers) of HSV-2 but not HSV-1 is sufficient for shutoff is the comparative potency at the level of specific activity of HSV-2 vhs. In addition to considerations of what governs the activity of input vhs early after infection, the result raises the possibility that other types of particles from an HSV-2 infection notably L-particles could, even via a single particle, contribute to transient shutoff of translation, in advance of or associated with infection by a complete infectious particle. Indeed it has previously been shown that vhs is incorporated into L-particles and that L-particles may exhibit vhs activity [78, 79]. It is interesting to compare conclusions from distinct techniques. In previous studies [78] of high multiplicity infections, using population analysis of protein synthesis by ^35^S-methinone radiolabeling/SDS-PAGE analysis, the results indicated that approximately 400 virion particles were required to induce at least some translational suppression (measured at 5 hr p.i.). In contrast our data indicate that a single particle, at least of HSV-2, can induce pronounced early shutoff which is then reversed. In this regard, it is conceivable that the formation of a localised zone of translational shutdown, contributed to by single infectious virions or L-particles, is important for the strong physiological role of vhs in pathogenesis [72, 83–89].

### Translation suppression and recovery in relation to eIF4H localisation

Our results also reveal a pronounced relocation of a cellular translation factor, eIF4H, from its mainly cytosolic localisation to a mainly nuclear localisation. This relocation was tightly linked to translational suppression. Not only was this relationship highly statistically significant, in that shutoff at the advancing front and eIF4H relocation were almost completely congruent, but eIF4H is also one of the cellular translation factors previously shown to directly interact with vhs in biochemical studies [41]. Classical biochemical studies have shown that vhs binds directly both to eIF4H and to eIF4A, an RNA helicase with which eIF4H itself interacts [40, 41, 90, 91]. These interactions are thought to underpin the recruitment of vhs to the larger eIF4F cap binding complex, with such recruitment helping explain the specificity of vhs RNase activity in the degradation of mRNAs versus non-mRNAs in vivo [40, 92–95]. However, the precise mechanism of vhs activity, target selectivity and associating factors remain to be fully identified [52, 65]. Of the translation factors analysed here by immunofluorescence, there was clear selectivity in the relocalisation of eIF4H. While we could not observe any distinct alteration in eIF4AII, it remains possible that e.g. subpopulations of eIF4AII or indeed other translation factors are also relocalised in a manner correlating with translational suppression. While these results do not imply a causal association between eIF4H relocalisation and vhs-dependent translational suppression, nevertheless it is reasonable to integrate our results with the biochemical analyses cited above. Such analyses, further comparing w/t and selected vhs mutants, suggest that eIF4F association correlates with vhs binding to eIF4A, rather than eIF4H. At the same time however, recruitment of active vhs to eIF4F is not of itself sufficient to selectively degrade mRNAs in vivo and binding to eIF4H is thought to be required [38, 40, 41, 90]. Moreover, siRNA mediated knockdown of eIF4H result in a decline in the activity of vhs in degrading host mRNA early after infection, independent evidence that eIF4H is specifically involved in vhs mRNA degradation and thus translational suppression [96].

We first considered that eIF4H relocation could be a general effect of infection, independent of vhs activity. However this was not the case, and relocation clearly required catalytically active vhs. Thus several not mutually exclusive explanations could account for the combined observations of on the one hand, a required interaction between vhs and eIF4H for mRNA degradation and on the other, eIF4H nuclear relocation tightly correlating with vhs-dependent translational suppression. Perhaps most plausible is the proposal that relocation is not causal, but is a downstream consequence of vhs activity. Once targeted by vhs for its recruitment to mRNA and after mRNA cleavage, eIF4H could be released from the RNase complex and then be somehow altered or lack a recycling/targeting mechanism for cytoplasmic retention. It could then (eIF4H is 248 residues long with a mol. wt of ca. 27kD) passively relocate to the nucleus and/or be retained there. An alternative explanation, but one also invoking relocation as a consequence of vhs function, would be some form of active shunting of the eIF4H cytoplasmic pool to the nucleus. While requiring vhs function, this would not exclude participation of other virus factors in relocation. Moreover, it appears that the majority of eIF4H is retained in the nucleus during translational recovery. This result may reflect a qualitative change in the nature of the translational apparatus during translational recovery, for example with eIF4H playing a different or dispensable role in the restoring infected cell. It could also be that this relocalisation reflects a broader change in the nature of the translational apparatus and the qualitative processes operating occurring during translational recovery compared to prior to shutoff. The precise explanation for the relocation of eIF4H and the consequences for later infected cell translation requires further investigation.

Finally, it is interesting to speculate on the implications of vhs mediated biphasic translational oscillation on its role(s) in vivo. Although vhs deletion does have an effect on replication, vhs is dispensable in tissue culture and deletion mutants replicate comparatively well [62, 83]. However vhs plays very significant roles in virulence and pathogenesis in vivo in various animal models [72, 83–85, 88, 97, 98]. It is becoming increasing clear that the loss of virulence and pathogenic outcomes of vhs mutants is due to an important role in immune evasion [reviewed in 52]. Proposals in this regard include vhs involvement in down regulation of MHC class I and II, suppression of the production of proinflammatory cytokines and blocking dendritic cell activation [99–102]. One attractive possibility therefore is that the oscillating nature of translational activity mediated by vhs is an integrated function, wherein the rapid suppression of translation is sufficient to compromise host cell functions, (e.g. protein delivery, immune cell activation) immediately after infection but where sustained translational suppression would impact on virus progression. Thus translation needs to be restored to provide necessary cellular de novo synthesised proteins, as well as viral proteins for progressive replication.

In conclusion, while considerable insight into metabolic processes during virus infection has been gained from classical molecular and biochemical analysis at the population level, information at the individual cell level is also critical for a true understanding of the processes governing the outcomes of infection. Such analyses cannot answer all questions of detailed mechanisms and cannot replace molecular and biochemical investigation of these processes. However, these techniques of bioorthogonal chemistry in combination with immunofluorescence, complement biochemical and population approaches and can reveal new insight not appreciated by classic techniques. Our results support previous data from biochemical analysis but also reveal new understanding from which we propose a revised model of translational control during advancing infection which has important implications both mechanistically and with regards to the physiological role of translational control during infection in vivo. This work opens avenues for future investigation of infected cell translation, the mechanisms involved in suppression, the function of vhs and the role(s) of other viral and host factors. It is also highly applicable to other virus systems.

## Materials and methods

### Cells and viruses

African Green Monkey kidney fibroblast (Vero) cells, obtained from the European Cell Culture Collection, Porton Down, UK) and human epithelial keratinocytes (HaCaT), obtained from Professor Gill Elliott, University of Surrey, UK, were grown in Dulbecco’s Modified Eagle’s Medium (DMEM; Gibco) supplemented with 10% Fetal Bovine Serum (FBS; Gibco), and penicillin/streptomycin (Gibco). The viruses used in this study were parental strains HSV-1[17], HSV-1[KOS] and the vhs mutant HSV-1[17].Δvhs [82] and for HSV-2, HSV-2[186] and mutants ΔUL41, ΔUL41-REP, D215N, and D215N-REP [58]. For studies during cell-cell transmission, single particle infections were routinely performed by infecting a confluent monolayer of cells (2x10^5^ cells) with 50 PFU with neutralising human serum added 1 hr post infection. HPG-pulse labeling intervals were initiated approximately 24 hr later. Studies during single step replication were performed at a multiplicity of infection (MOI) of 10. Particle/pfu ratios of selected virus stocks (routinely a ratio of approximately 50) were determined as previously described [103]. To analyse translation at the earliest times possible and in as synchronised a manner as possible (see e.g., Fig 3), cells were infected at +4°C for 1 hr, shifted to 37°C for 45 min to allow infection to proceed and 45 min later methionine depleted and pulse-labeled with HPG for 30 min. Omitting a methionine depletion stage or shortening the labeling interval in the attempt to analyse translation at even earlier times resulted in a vastly reduced signal. The protocol adopted was the optimal compromise for sensitive labeling at the earliest time possible. For analysis of the earliest stages of single particle infection, infections were performed at MOIs of 0.1, 0.01 and 0.005 in methionine depleted medium, and the cultures then incubated in labeling medium for 30 min as described.

### Antibodies for immunofluorescence studies

The following antibodies were used: mouse anti-ICP4 MAb (Virusys, 1:500); mouse anti-VP5 MAb (Virusys, 1:200); mouse anti-gB MAb (Sigma-Aldrich,1:100); mouse anti-protein disulfide isomerase (PDI) Mab (Abcam, 1:50); mouse anti-eIF4H Mab (Santa Cruz, 1:50); mouse anti-eIF2α Mab (Santa Cruz, 1:50); mouse anti-eIF4AII Mab (Santa Cruz, 1:50); mouse anti-eIF4B Mab (Santa Cruz, 1:50); mouse anti-eIF4G Mab (Santa Cruz, 1:50); and Alexa Fluor 594 goat anti-mouse IgG (Molecular Probes, 1:500).

### Immunofluorescence studies

For immunofluorescence analysis, cells on glass coverslips were fixed at times indicated in 4% paraformaldehyde (Pierce) for 10 min, permeabilised with 0.5% Triton X-100 (Sigma) for 5 min, and blocked with phosphate-buffered saline (PBS; Sigma) containing 10% FBS for 30 min at room temperature (RT). Cells were immunolabeled for 1 hr at RT with primary antibodies and 45 min with secondary antibodies, followed by click chemistry and mounting in ProLong Gold Antifade Mountant (Molecular Probes). Images were with a Zeiss Axiovert 135 TV microscope system using Zeiss 63x (Plan-APOCHROMAT, 1.4 numerical aperture), 40x (Plan-Neofluar, 0.75 numerical aperture), or 20x (Plan-Neofluar, 0.5 numerical aperture) objectives and a Retiga 2000R camera with Image Pro Plus 7.0 software. Alternatively, images were acquired with a Zeiss Laser Scanning Confocal Microscope system using 488 nm and 543 nm lasers with Zeiss LSM 5 software. Each channel was collected separately, with images at 1024 x 1024 pixels, with 4x averaging and without or with a variable zoom factor. Single confocal sections were acquired or multiple z-sections at 1 μm intervals which were then compiled for maximum projection display. For all low moi early infected cell foci or plaques were randomly inspected for the presence of zones exhibiting regional shutoff at the periphery of the plaque. When comparing viruses, e.g. w/t versus a vhs mutant all plaques within a virus type showed an identical phenotype with regard to the presence or absence of such zones. For qualitatively or quantitatively assessing the extent of shutoff, as explained in the text (pg 11), inherent asynchrony in infection and other variables, means that there was not a continuous zone of shutoff around the developing plaque. Rather there are regional zones with defined features of pronounced translational suppression within and between areas of normal translation, where the inner part of the zone was at the edge of a distinct infected area, (defined by virus antigen or morphology) Selected representative fields exhibiting such zonal shutoff were examined for translation (by click chemistry, see below), antigen presence (by immunofluorescence) and cell numbers (by DAPI and phase). Usually at least 10 fields were specifically evaluated and imaged using x40 or x63 lenses, (approximately 120 cells and 40 cells on average per field respectively). Thus for representative images, each panel in a figure is a representative image of a zone from about 50 plaques, 10 fields, and 400–500 cells.

### Homopropargylglycine (HPG) pulse-labeling and click chemistry

From systematic analysis of the HPG concentration and duration of pulse, we optimised protocols for HPG incorporation, click chemistry and fluorescence detection as follows. Cells on coverslips were mock-infected or infected with HSV by standard procedures. At times indicated, medium was removed and replaced with L-methionine-free DMEM (Sigma-Aldrich) containing 2% FBS for 45 min to deplete methionine prior to the addition of HPG (Molecular Probes) at a final concentration of 0.5 mM for an optimised labeling time of 30 min in L-methionine-free DMEM. When the pulse-labeled cells were to be analysed in parallel for localisation of specific antigens, immunofluorescence with primary and secondary antibodies was carried out as standard (see above). Samples were then subjected to click reaction in a buffer freshly prepared in each case (premixed for 2 min) and containing 10 μM Alexa Fluor 488-azide (Sigma); 1 mM CuSO_4_; 10 mM sodium ascorbate; 10 mM amino-guanidine and 1 mM Tris(3-hydroxypropyltriazolylmethyl)-amine (THPTA; Sigma-Aldrich) in PBS pH 7.4. The reaction was allowed to proceed by incubation for 2 hr at RT in the dark. After removal of the reaction cocktail, cells were washed with PBS and mounted on slides in ProLong Gold Antifade Mountant. Images were acquired as described above.

### Quantitative analysis of HPG intensity

For quantitation of the global protein synthesis at the single cell level, we used the thresholding and object quantification modules of Image Pro Plus software (Media Cybernetics). We evaluated several routes for individual cell quantification. We found that the cytoplasm of cells frequently overlapped between adjacent cells (observed better by HPG incorporation than by phase microscopy). Moreover, the nuclear signal represented a considerable fraction of the total. Considering these features, we therefore used the nuclear signal as a measurement of relative levels of protein synthesis between cells. While not measuring total level of protein synthesis, this parameter give a more accurate measurement of between-cell variations within populations than attempting to delineate entire cell boundaries. HPG-labeled cells were co-stained with DAPI to allow outlining of the entire nucleus and creation of a mask for each field. The masks were applied onto the HPG-green channel in which the HPG intensity of each individual cell (tagged as objects) was quantitated and normalised to nuclear area. To delineate the cells within different zones (distal uninfected cells, shutoff zone and interior infected focus zone), we set a threshold for significant suppression to be at 30% or below of the maximum intensity for the field and coded this zone as the shutoff zone (yellow). Cells having intensities above this threshold were coded pink. This method is somewhat conservative since translation levels in uninfected cells (i.e. in mock-infected monolayers) were relatively consistent, rarely exhibiting levels below 50–60% of the maximum in the field. Setting the threshold at 30% may therefore miss cells that were in partial shutoff but this does not materially alter our conclusions.

### Click chemistry and in-gel fluorescence of newly synthesised proteins

Cells were mock-infected or infected with HSV by standard procedures. In control experiments, cells were incubated with HPG in the absence or presence of 100 μg/ml cycloheximide (CHX), added 1 hr before methionine depletion (S1b Fig). Cells were lysed in PBS containing 2% SDS and diluted to 1% SDS before the click reaction. 100 μg of protein samples were subjected to the click reaction as follows. Click reaction buffer consisting of the capture reagent (0.1 mM IRDye 800CW Azide Infrared Dye from LI-COR); 1 mM CuSO_4_, 2 mM Tris-(2-Carboxyethyl)phosphine (TCEP; Sigma-Aldrich), 0.2 mM Tris(benzyltriazolylmethyl)amine (TBTA; Sigma-Aldrich) was freshly prepared. Following the addition of the click mixture, samples were placed on a rotating mixer for 1.5 hr at RT, and the reaction was stopped by addition of EDTA to a final concentration of 10 mM. Subsequently, proteins were precipitated (chloroform/methanol, 0.25:1, relative to the sample volume). The precipitated proteins were pelleted by centrifugation at 14,000 rpm for 5 min, washed with methanol and air dried for 10 min. The pellets were then resuspended in 1x SDS sample buffer, boiled for 5 min, and 20 μg of proteins were loaded on 12% SDS-PAGE gels. Following electrophoresis, gels were washed with water, fixed in solution containing 40% methanol, 10% acetic acid, 50% water for 5 min and washed with water. In-gel fluorescence detection of translated proteins was performed using a LI-COR Odyssey infrared imaging system, and the protein loading was assessed by Coomassie blue staining.

## Supporting information

S1 Fig Biochemical and spatial analysis of translation using click chemistry. (A) Schematic diagram illustrating comparative structures of methionine and HPG. The scheme indicates the in vivo incorporation of HPG into protein (solid black dots within a protein chain) and then the subsequent in vitro cycloaddition reaction to covalently cross link an azide fluorochrome-coupled capture reagent (coloured star) to HPG. (B) In optimising HPG labeling parameters, we pulsed cells with 1 mM HPG for 30 min at 1 hr after mock infection or infection with HSV-1 (MOI 10). Cells were lysed and subjected to click reaction using IRDye 800CW Azide Infrared Dye. Proteins were then separated by SDS-PAGE and visualised by in-gel fluorescence (green) using a LI-COR Odyssey Infrared Imaging System. As a control we included cycloheximide (CHX, 100 μg/ml) to block de novo protein synthesis. Total protein content is shown in lanes 1–4 and the same gel scanned for de novo synthesised proteins lanes 4–8. The results demonstrate efficient labeling of uninfected cell proteins (lane 7) with essentially no significant change in overall levels of translation in HSV infected cells at this early time (c.f. lanes 7 and 8). In the presence of CHX, incorporation was virtually eliminated (c.f., lanes 5 and 7 or 6 and 8). (C) Using a 30 min labeling interval as a benchmark we then labeled cells for progressively shorter or longer intervals to assess the appropriate interval in terms of sensitivity and dynamic range. Cells were fixed and subjected to click reaction using Alexa Fluor 488-azide (green channel) combined with simultaneous immunofluorescence using the ER marker PDI (red). The results demonstrated that while newly translated proteins could be visualised with an interval as short as 5 to 10 min, the sensitivity and dynamic range were somewhat limited. Extending the interval 30 min revealed efficient incorporation and labeling of proteins seen throughout cytoplasmic compartments including the ER and distinct accumulation in the nucleus and nucleolus (see also Fig 1). Longer labeling intervals exhibited somewhat increased new protein accumulation but 30 min was selected as the standard labeling interval, exhibiting a very distinctive difference from background levels in the absence of HPG and a very good dynamic range.
(TIF)

S2 Fig Cell type modulation of the efficiency of regional shutoff. (A) Vero or HaCaT cells were infected (MOI 0.0005) with HSV-1[KOS] according to the standard workflow in Fig 1b, and analysed for newly synthesised proteins (green) and VP5 accumulation (red). (B) HaCaT cells were infected as above and HPG pulse-labeled at 25 hr p.i. and 50 hr p.i.
(TIF)

S3 Fig Analysis of localisation of candidate translation factors in relation to translational suppression. Vero cells were infected with HSV-2[186] at a MOI 0.0005 according to the standard workflow and analysed for newly synthesised proteins (green) and localisation of a series of translation factors as indicated (red). Representative images at the periphery of the advancing infection showing cells exhibiting pronounced translational suppression (cells numbered 1) adjacent to distally located cells (i.e., external to the origin of developing plaque), where there was no shutoff (cells numbered 2). No discernible difference could be observed for each of these factors in the two situations.
(TIF)
