# Supplementary figures and images for: A bimodal switch in global protein translation coupled to eIF4H relocalisation during advancing cell-cell transmission of herpes simplex virus
Source: PLoS Pathog. 2018 Jul 20;14(7):e1007196. doi: 10.1371/journal.ppat.1007196 (PMC6070287; doi:10.1371/journal.ppat.1007196)

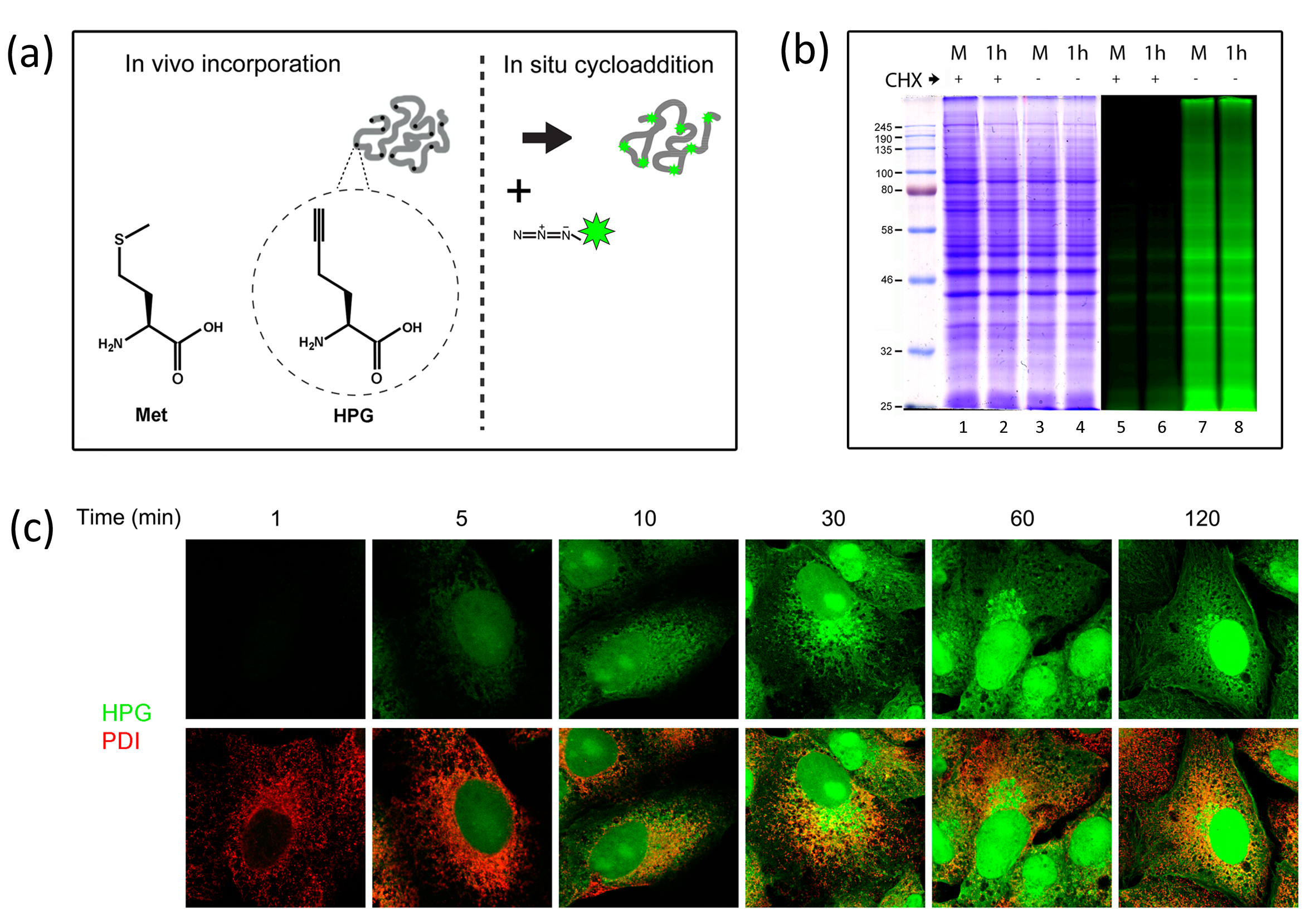

Supplement: S1 Fig — (A) Schematic diagram illustrating comparative structures of methionine and HPG. The scheme indicates the in vivo incorporation of HPG into protein (solid black dots within a protein chain) and then the subsequent in vitro cycloaddition reaction to covalently cross link an azide fluorochrome-coupled capture reagent (coloured star) to HPG. (B) In optimising HPG labeling parameters, we pulsed cells with 1 mM HPG for 30 min at 1 hr after mock infection or infection with HSV-1 (MOI 10). Cells were lysed and subjected to click reaction using IRDye 800CW Azide Infrared Dye. Proteins were then separated by SDS-PAGE and visualised by in-gel fluorescence (green) using a LI-COR Odyssey Infrared Imaging System. As a control we included cycloheximide (CHX, 100 μg/ml) to block de novo protein synthesis. Total protein content is shown in lanes 1–4 and the same gel scanned for de novo synthesised proteins lanes 4–8. The results demonstrate efficient labeling of uninfected cell proteins (lane 7) with essentially no significant change in overall levels of translation in HSV infected cells at this early time (c.f. lanes 7 and 8). In the presence of CHX, incorporation was virtually eliminated (c.f., lanes 5 and 7 or 6 and 8). (C) Using a 30 min labeling interval as a benchmark we then labeled cells for progressively shorter or longer intervals to assess the appropriate interval in terms of sensitivity and dynamic range. Cells were fixed and subjected to click reaction using Alexa Fluor 488-azide (green channel) combined with simultaneous immunofluorescence using the ER marker PDI (red). The results demonstrated that while newly translated proteins could be visualised with an interval as short as 5 to 10 min, the sensitivity and dynamic range were somewhat limited. Extending the interval 30 min revealed efficient incorporation and labeling of proteins seen throughout cytoplasmic compartments including the ER and distinct accumulation in the nucleus and nucleolus (see also Fig 1). Longe [file ppat.1007196.s001.tif]

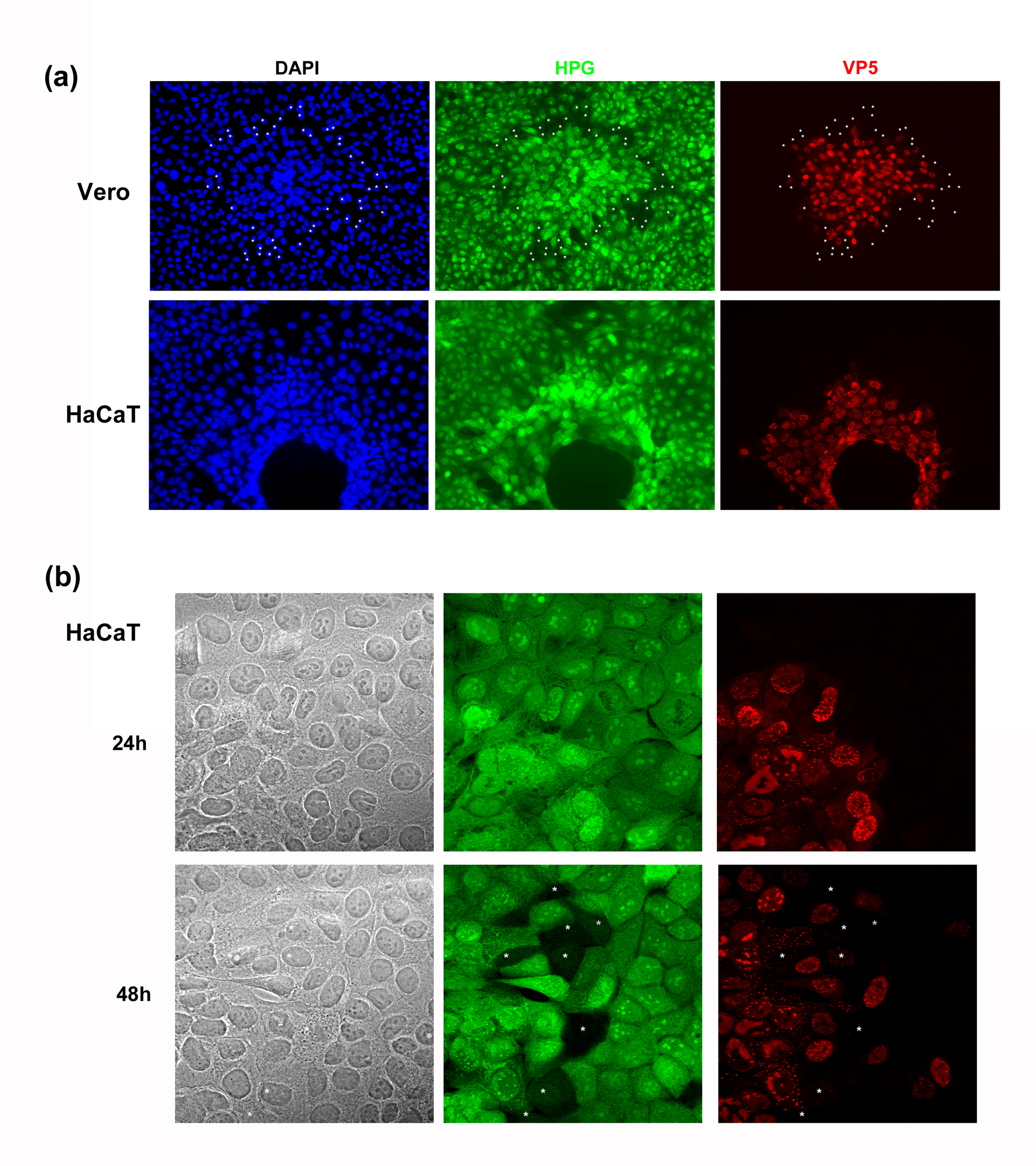

Supplement: S2 Fig — (A) Vero or HaCaT cells were infected (MOI 0.0005) with HSV-1[KOS] according to the standard workflow in Fig 1b, and analysed for newly synthesised proteins (green) and VP5 accumulation (red). (B) HaCaT cells were infected as above and HPG pulse-labeled at 25 hr p.i. and 50 hr p.i. (TIF) [file ppat.1007196.s002.tif]

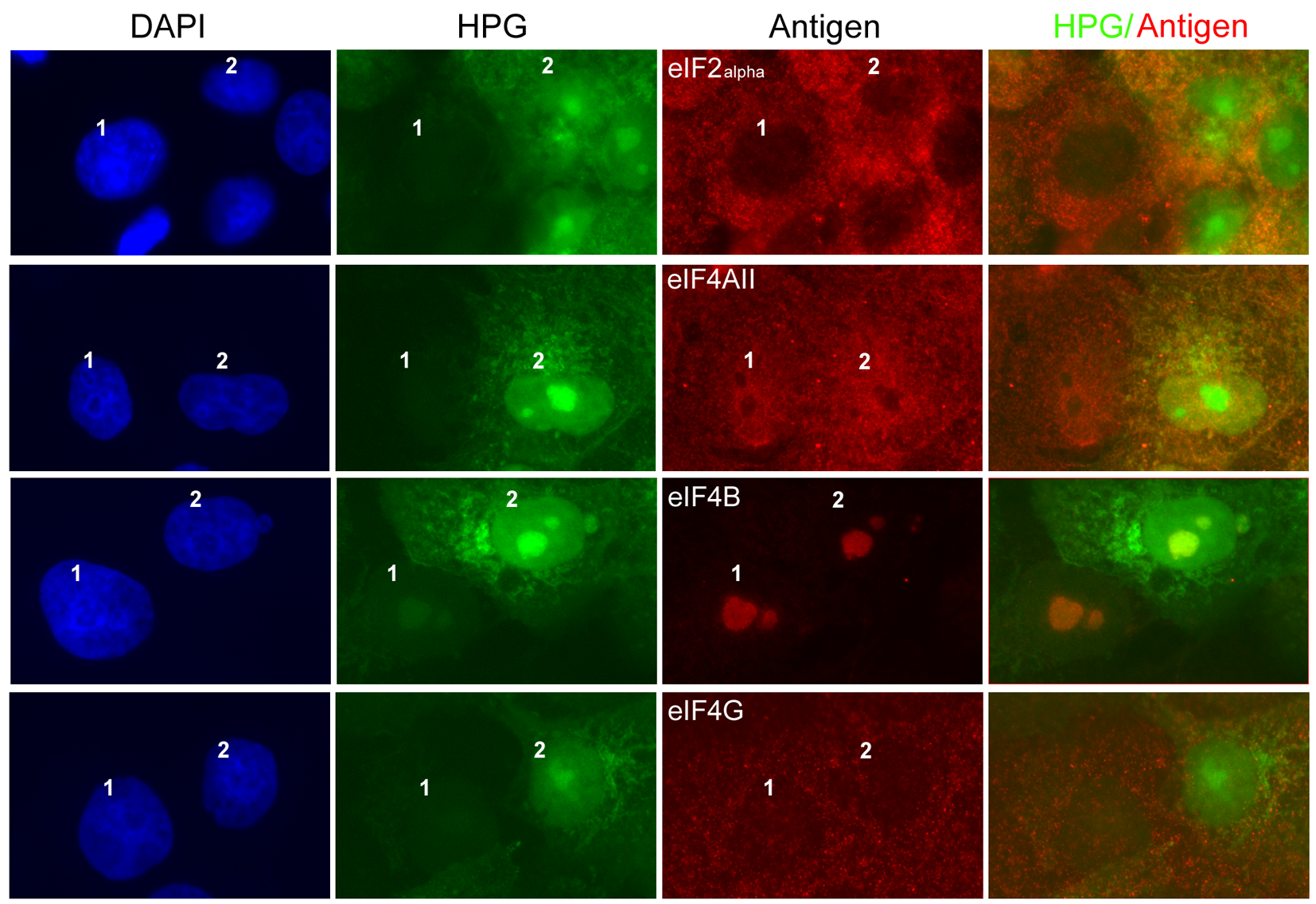

Supplement: S3 Fig — Vero cells were infected with HSV-2[186] at a MOI 0.0005 according to the standard workflow and analysed for newly synthesised proteins (green) and localisation of a series of translation factors as indicated (red). Representative images at the periphery of the advancing infection showing cells exhibiting pronounced translational suppression (cells numbered 1) adjacent to distally located cells (i.e., external to the origin of developing plaque), where there was no shutoff (cells numbered 2). No discernible difference could be observed for each of these factors in the two situations. (TIF) [file ppat.1007196.s003.tif]
